# Mediterranean Diet and Oxidative Stress: A Relationship with Pain Perception in Endometriosis

**DOI:** 10.3390/ijms241914601

**Published:** 2023-09-27

**Authors:** Michela Cirillo, Flavia Rita Argento, Matteo Becatti, Claudia Fiorillo, Maria Elisabetta Coccia, Cinzia Fatini

**Affiliations:** 1 Department of Experimental and Clinical Medicine, University of Florence, 50134 Florence, Italy; cinzia.fatini@unifi.it; 2 Centre for Assisted Reproductive Technology, Division of Obstetrics and Gynecology, Careggi University Hospital, 50134 Florence, Italy; mariaelisabetta.coccia@unifi.it; 3 Department of Clinical and Experimental Biomedical Sciences, University of Florence, 50134 Florence, Italy; flaviarita.argento@unifi.it (F.R.A.); matteo.becatti@unifi.it (M.B.); claudia.fiorillo@unifi.it (C.F.)

**Keywords:** lifestyle, diet, endometriosis, chronic pelvic pain, pain management, inflammation

## Abstract

Background: Endometriosis is a chronic and inflammatory disease associated with pelvic pain. Dietary changes may be therapeutic for chronic inflammatory processes, reducing visceral input. The aim was to evaluate the role of dietary changes according to the Mediterranean Diet (MD) on pain perception in endometriosis and their relationship with oxidative stress. Methods: in this prospective study, we included 35 endometriosis women. At baseline (T0) and after 3 (T1) and 6 (T2) months from the start of the diet, we investigated pain intensity with VAS (Visual Analogue Scale, from 0 to 10), vitamin profile, and oxidative stress. Results: we found a significant increase in the diet score (*p* < 0.001). At T1, patients reduced pain in terms of dyspareunia (*p* = 0.04), non-menstrual pelvic pain (*p* = 0.06), dysuria (*p* = 0.04), and dyschezia (*p* < 0.001). Dyspareunia (*p* = 0.002) and dyschezia (*p* < 0.001) were further significantly reduced also at T2. We observed a significant positive correlation between lipid peroxidation and VAS non-menstrual pelvic pain and dysuria and a significant negative correlation between Oxygen radical absorbance capacity and VAS non-menstrual pain and dyschezia. Conclusions: our findings show a clear tendency toward a relationship between pain relief in endometriosis and MD. This appears promising to treat endometriosis-related symptoms and could be considered a new effective strategy for chronic pain management in the long term.

## 1. Introduction

Endometriosis is a chronic, inflammatory, debilitating disease associated with pelvic pain and infertility, characterized by endometrial-like tissue outside the uterus [1]. Endometriosis affects roughly 10% (190 million) of reproductive-age women and girls globally.

Based on different populations and diagnostic tools, the estimate varies. Indeed, the prevalence is roughly 2–11% in asymptomatic, 5–50% among infertile and 5–21% in women hospitalized for pelvic pain. The prevalence of endometriosis among symptomatic adolescents ranges from 49% (when chronic pelvic was present) to 75% when pain was unresponsive to pharmaceutical treatment [2].

The clinical presentation of endometriosis is complex. Symptomatically, it includes several features yet non-specific types of pain, an increased risk of infertility, fatigue, exhaustion, headache, intestinal discomfort, and lack of energy.

Pelvic pain is a cardinal symptom for many individuals suffering from endometriosis, but it is not a specific indicator since it is associated with several gynecological and non-gynecological disorders. A progressive dysmenorrhea should raise suspicion for endometriosis since primary dysmenorrhea does not increase in severity over time.

Pain perception can vary individually in intensity, location, time of occurrence and duration.

Endometriosis is a significantly heterogeneous disease, both in phenotype and clinical outcomes, that differs from no symptoms to severe pain and/or subfertility, leading to a significant negative impact on women’s quality of life [3,4].

According to the results of a systematic review, including mainly randomized controlled trials, at least 10% of patients had no benefits in pain reduction with pharmaceutical treatment, and up to 60% of them reported pain when the treatment was stopped [5]. Nociceptors are activated by the chronic inflammatory state with the result of central and peripheral sensitization: this appears to be linked with referred pain due to the mechanism induced by the noxious visceral input via the spinal cord, thus sensitizing multiple areas with consequent hyperalgesia, allodynia and referred pain [6].

No definitive cure exists for endometriosis; treatments consist of hormonal medication, surgical removal of lesions, or both, often with side effects and suboptimal efficacy [7]. Many women feel the need to take control and assist in managing their chronic disease themselves and possibly increasing their quality of life. Therefore, promoting both primary and secondary prevention and implementing treatment strategies which address all patient needs, including quality of life issues, are necessary [8].

Over the last decade, studies to examine the role of diet in endometriosis have gained more attention since it has been observed that diet can affect several processes involved in endometriosis, such as inflammation, prostaglandin metabolism and estrogen activity [9]. Some studies suggested that dietary changes may be therapeutic for chronic inflammatory processes, thereby reducing visceral input [10,11,12]. In this context, the Mediterranean dietary pattern, rich in fruits, vegetables, legumes, and whole grains, represents an evidence-based nutritional model for preventing cardiovascular incidence and all causes of mortality and a possible non-pharmacological therapeutic intervention in endometriosis.

To date, it is evident that this topic is characterized by an extreme paucity of scientific data and an extreme dishomogeneity of the results obtained. The study aimed to evaluate the role of dietary changes according to the Mediterranean Diet (MD) pattern on pain perception in women with endometriosis and their relationship with oxidative stress.

## 2. Results

### 2.1. Mediterranean Diet and Pain Perception

Thirty-five participants completed the first phase (T1), and 26 women the entire study (T2). The baseline demographic and clinical characteristics of the population studied were previously published [13].

**Figure 1:**
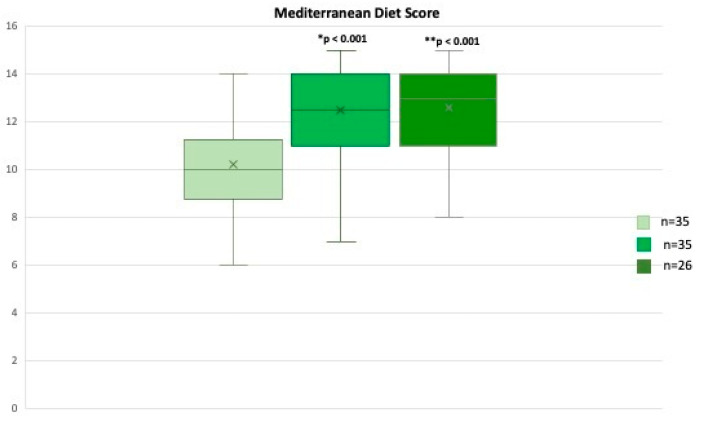
Mediterranean Score adherence (scores range from 0 to 18) from T0 to T2. * T0 vs. T1, ** T0 vs. T2. Values are expressed as median (range).

Through the analysis of the MD adherence score (scores range from 0 to 18) at the end of the first (T1) and second phases (T2), we found a statistically significant increase in the median adherence score (T0 vs. T1, *p* < 0.001) (Figure 1), that was still significantly higher also at T2 with a median value of 13 (8–15), (T0 vs. T2, *p* < 0.001) (Figure 1).

**Figure 2:**
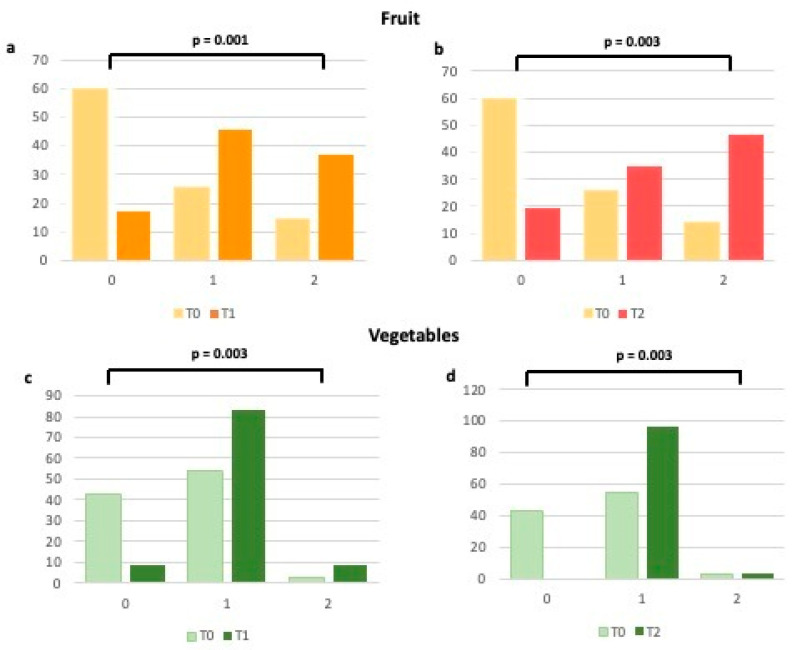
Changes in adherence to the Mediterranean Diet (expressed as percentages) for fruit and vegetable consumption from T0 to T1 (**a**,**c**) and from T0 to T2 (**b**,**d**). 0 = 0 points at the Mediterranean adherence score; 1 = 1 point at the Mediterranean adherence score; 2 = 2 points at the Mediterranean adherence score.

**Figure 3:**
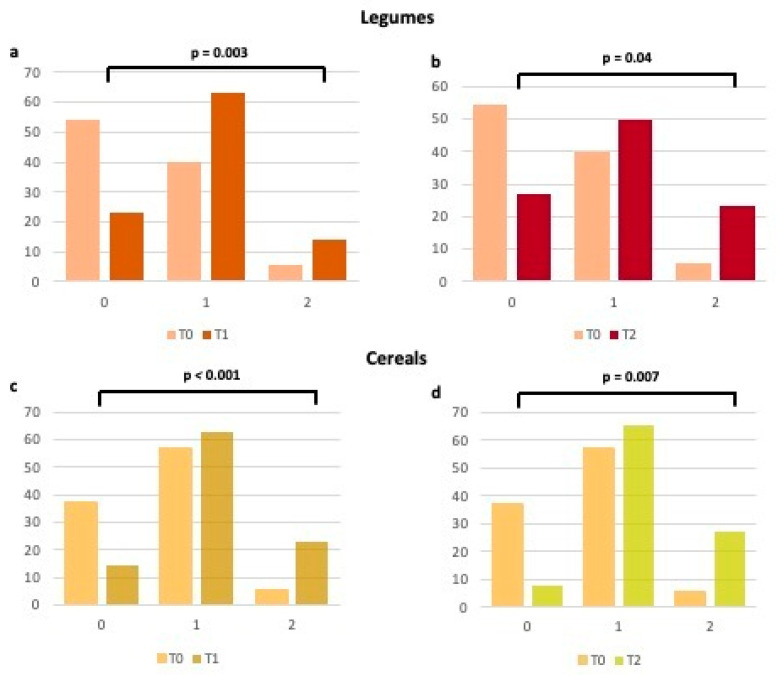
Changes in adherence to the Mediterranean Diet (expressed as percentages) for legumes and cereals consumption from T0 to T1 (**a**,**c**) and from T0 to T2 (**b**,**d**). 0 = 0 points at the Mediterranean adherence score; 1 = 1 point at the Mediterranean adherence score; 2 = 2 points at the Mediterranean adherence score.

**Figure 4:**
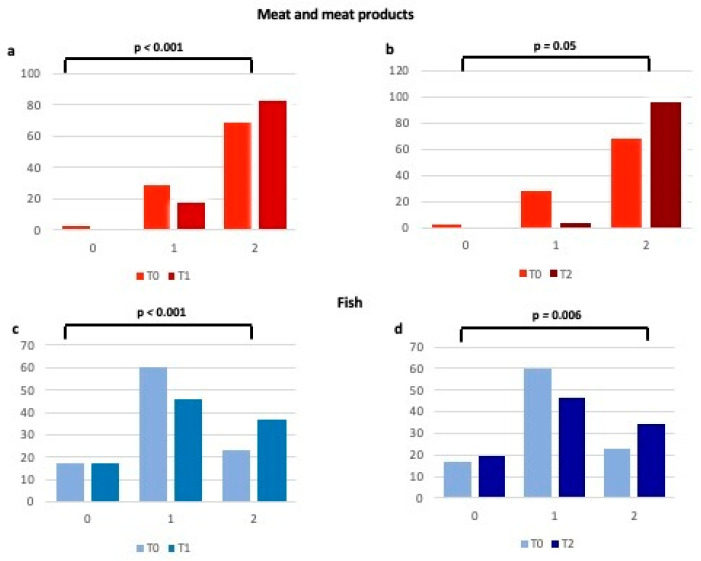
Changes in adherence to the Mediterranean Diet (expressed as percentages) for meat and meat products and fish consumption from T0 to T1 (**a**,**c**) and from T0 to T2 (**b**,**d**). 0 = 0 points at the Mediterranean adherence score; 1 = 1 point at the Mediterranean adherence score; 2 = 2 points at the Mediterranean adherence score.

When analyzing each item of the validated questionnaire [14,15], we observed how much higher the number of patients who have enhanced their consumption of fruits, vegetables, legumes, and cereals (Figure 2 and Figure 3), typical foods of the MD at T1 and T2 in comparison to T0. In particular, the percentage of women with a score raising from 0 (poor consumption) to 1 (moderate consumption) for each of the above items increased at T1, thus remaining substantially unchanged at T2 (Figure 2 and Figure 3). Regarding meat and processed meats (Figure 4), it is worth noting that, even if adherence at T0 was already high, it increased further at T1, particularly due to the limited consumption of red meat, as stated by the patients. Regarding fish consumption (Figure 4), many women indicated that they increased their intake to more than 2–3 times a week at both T1 and T2. However, 17.1% said they did increase fish consumption, probably due to not liking it or the cost.

The consumption of extra virgin olive oil was regular in 100% of the patients from baseline visit onwards, without any worsening through T0 to T2.

**Figure 5:**
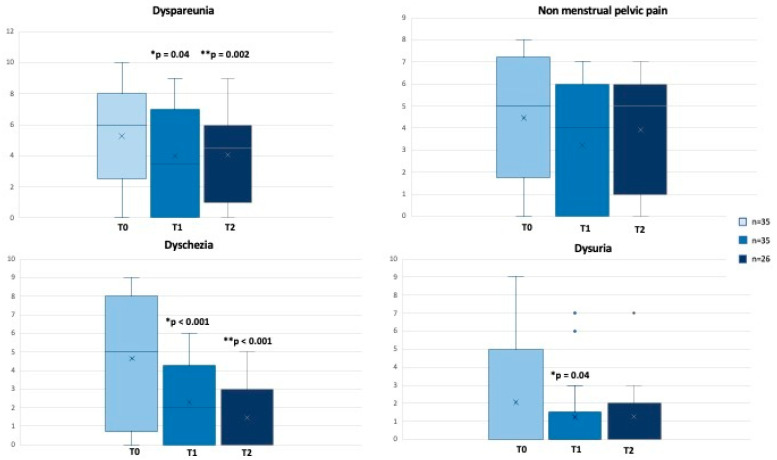
Changes in VAS Pain (scores range from 0 to 10) from T0 to T2. * T0 vs. T1, ** T0 vs. T2. Values are expressed as median (range).

At the same time, besides the dietary changes, we evaluated pain perception from T0 to T2. At T1, patients reduced pain in terms of dyspareunia (*p* = 0.04), non-menstrual pelvic pain (*p* = 0.06), dysuria (*p* = 0.04), but mainly dyschezia (*p* < 0.001) (Figure 5). Dyspareunia and dyschezia were further significantly reduced at T2 (*p* = 0.002 and *p* < 0.001, respectively) (Figure 5).

Furthermore, we found a modification in bowel habits (regarding changes from constipation or diarrhoea to a better regularity in frequencies and stool consistency). While at T0, 51.4% of the patients reported difficulties in the evacuation with altered stool consistency, at T1, a statistically significant improvement was found in about 35% (*p* = 0.002). This may have influenced VAS regarding dyschezia (higher intake of water and fiber, and more physical activity, as previously described) [13].

At T2, by analyzing the correlation between MD score and pain perception, we observed a significantly negative correlation with VAS dyschezia (rho = −0.499, *p* = 0.009).

### 2.2. Oxidative Stress, Mediterranean Diet and Pain Perception

We evaluated blood global redox status by assessing leucocyte intracellular ROS production, plasma lipid peroxidation, and plasma total antioxidant capacity, and we did not observe significant changes between T0 and T1 but an improvement between T0 and T2, as previously published [13].

Our previous study [13] reported that women with endometriosis had significant alterations compared to the normal range of oxidative stress parameters observed in a population of healthy age-matched women. In the present study, we focused the attention on the relationship between oxidative stress and pain perception (Table 1). In particular, we observed a significant positive correlation between lipid peroxidation and VAS non-menstrual pelvic pain and dysuria, and a significant negative correlation between ORAC and VAS non-menstrual pain and dyschezia (Table 1), as well as a significant positive correlation between ROS and dysuria.

Indeed, we considered the relation between adherence to the Mediterranean dietary pattern and oxidative stress, and we observed at T2 a significant negative correlation between ROS production and MD score (Neutrophil-ROS: rho = −0.55, *p* = 0.03; Monocyte-ROS: rho = −0.66, *p* = 0.007; Lymphocyte-ROS: rho = −0.55, *p* = 0.03). Based on this observation, we also evaluated the vitamin profile and observed an increase in B12, vitamin E, folate, and zinc (Table 2). In particular, we found a statistically significant increase in blood levels of folate from T0 to T2 (*p* = 0.006) (Table 2), which negatively correlated with Lymphocyte-ROS (rho = −0.59, *p* = 0.002) and Monocyte-ROS (rho = −0.63, *p* = 0.01).

## 3. Discussion

The results of our study show a clear tendency toward a relationship between pain relief in endometriosis patients, which included non-menstrual pelvic chronic pain, dyspareunia, dysuria and dyschezia, and Mediterranean dietary patterns. Our results align with Ott et al. in demonstrating that MD might lead to symptom relief in patients with endometriosis [11]. The Mediterranean dietary pattern may improve endometriosis-associated pain symptoms via various mechanisms, such as an anti-inflammatory effect. In particular, fish rich in omega-3 fatty acids and extra virgin olive oils were recommended because of their anti-inflammatory effect. In addition to the anti-inflammatory effect, the composition of the diet was also designed to have an eupeptic effect. In our study, before the dietary intervention, the daily fiber intake in women with endometriosis was below the recommendations [13].

Therefore, in our dietary intervention, we increased fiber intake through high consumption of fruit, vegetables, and whole grains, to shorten the transit time through the intestinal tract and to control the fecal excretion of excess estrogen. We know well that fecal excretion of excess estrogen is determined by increased fiber intake, while elevated serum estrogen levels related to endometriosis favor prostaglandin production, contributing to inflammation and proliferation of the disease [16]. Moreover, evidence from the literature shows that when the subjects have better adherence to the MD, they have a greater presence of Bacteroidetes, higher counts of Bifidobacterial, and higher levels of SCFA (short-chain fatty acids), the main metabolites produced in the colon by bacterial fermentation of dietary fibers and resistant starch. These results are consistent with a human study in which the authors observed that, after a 3-month MD intervention, the subjects had a significant change in their gut microbiome composition [17].

Many important points related to linkages between diet and endometriosis are worth noting. As a starting point, dietary-associated factors with well-known anti-inflammatory properties may mitigate the progression of the disease. This has been assessed as lesion size in murine models and/or lower risk for the advanced stage of endometriosis and/or reduction in the level of pain in endometriosis.

The different dietary factors evaluated in many previous experimental animal and human studies (e.g., green tea, resveratrol, soy isoflavones, fish oils) have shown health benefits. Thus, their ability to alleviate endometriosis is not entirely surprising. Santanam et al. evidenced in women with pelvic pain and endometriosis or infertility a significant reduction in peritoneal fluid inflammatory markers compared to patients not taking vitamins [18]. Second, women with endometriosis and experimental models respond similarly to dietary factors with anti-inflammatory effects.

Lastly, fish oils, PUFA, vitamins and flavonoids as possibly emerging candidates in endometriosis pain management and the reduction of lesion size, are in line with earlier reports from dietary recall questionnaires that suggested a negative link between green vegetables, fish oils, fruits and dairy products and the risk of endometriosis [19,20,21]. PUFA significantly affects the synthesis and activity of cytokines (IL-1, IL-6, TNF-α) and prostaglandins. Moreover, in the dynamic structures of the cell membrane, PUFA synergically interacts with phospholipids and antioxidants in steading and increasing membrane fluidity, thus improving its biochemical performance [22].

Moreover, the results from the Nurses’ Health Study II evidenced that the consumption of red meat seems to increase pain symptoms. Then, this could manifest as an increase in the incidence of an endometriosis diagnosis. One possible mechanism is the effect of red meat on steroid hormones [23].

In an earlier published study with a cross-over design conducted in women with dysmenorrhea, a low-fat vegetarian diet increased sex hormone binding globulin (SHBG) levels and reduced pain [24]. This suggests a possible pathway through which red meat, which was shown to reduce SHBG concentrations and increase estradiol, may benefit women with endometriosis suffering from pain [25].

Pain in women with endometriosis is related to the response to tissue injury and the resulting inflammatory process and oxidative stress. Oxidative stress is a double-edged sword: in physiological processes, it serves as key signal molecules, while in endometriosis, it has a role in pathological mechanisms. Oxidative stress induces a cytotoxic effect, increased cell membrane permeability, enzymatic activation, DNA damage and cell death [26]. Based on this observation, in this study, we demonstrated a significant correlation between lipid peroxidation, ROS production and pain intensity, evaluated with VAS. On the other hand, we observed a negative correlation between ORAC, expression of antioxidant capacity, and pain intensity. Data from Sesti F. et al. [22] suggested that in women with chronic pelvic pain, there is an increase in lipid peroxidation markers and an alteration in antioxidant levels.

To date, several studies have evidence that higher intakes of fruit and vegetables, which are typical of the Mediterranean Diet and are rich in antioxidants, improve human health and fight free radical damage [27,28]. After three and six months of dietary intervention, we observed higher levels of vitamin profile, in particular folate, which is an effective free radical scavenger. Interestingly, higher levels of folate at T2 significantly and negatively correlated with lymphocyte and monocyte ROS production. We know that folate can protect bio-constituents from free radical damage, at least by competition, which otherwise can lead to oxidative stress. Despite being a water-soluble molecule, folate can inhibit lipid peroxidation also [29]. Moreover, we observed an increased level of B12 vitamin, E vitamin and Zinc. The latter are intracellular signaling molecules with anti-inflammatory properties and play a role in oxidative stress and immune functions, inhibiting free radicals’ production [30].

Moreover, in our previous study [13], we evidenced that the Mediterranean Diet improved the cardiovascular risk profile of women with endometriosis by modulating common mechanisms shared by atherosclerosis and endometriosis, such as proatherogenic lipid profile. This represents another healthy effect of the Mediterranean Diet since the development of a less atherogenic LDL phenotype could be a possible explanation for some of the cardioprotective benefits of this dietary pattern [13].

Our study shows that the improvement in vitamin blood levels is due to Mediterranean dietary changes, and their antioxidant activity can improve endometriosis-related pain symptoms.

Some limitations should be mentioned. Studies with a larger population and a longer duration are necessary to confirm these intriguing results, with a follow-up time of 12 months and annually for as long as possible. Moreover, in our study, all women were Caucasian. Therefore, the finding may not be generalizable to other racial and ethnic groups. However, notwithstanding these limitations, our study has several strengths. This is a prospective study, and we included women with confirmed diagnoses of endometriosis; moreover, we re-evaluated adherence to the MD in follow-up time, thus permitting us to monitor the effect of dietary intervention. Currently, limited evidence on the effect of diet on pain perception in women with endometriosis is provided, and our study may represent a piece of knowledge in the endometriosis puzzle.

## 4. Materials and Methods

In this prospective study, we evaluated, from March 2020 to December 2022, 90 Caucasian women with endometriosis referred to the Internal Medicine Clinic at the Assisted Reproductive Technology Center (Division of Obstetrics and Gynecology) of Careggi University Hospital, Florence, Italy. The diagnosis of endometriosis was confirmed by diagnostic imaging (US or MRI) and/or laparoscopy performed by a gynecologist at the Endometriosis Center of Careggi University Hospital, a third-level center for endometriosis treatment. Inclusion and exclusion criteria were previously published [13]. Therefore, in this study, we included 35 women with endometriosis who are of reproductive age, as previously published [13]. At baseline, all women were under estrogen-progestins or progestins therapy. In comparison to our previous study [13], 26 women completed the second phase of the dietary intervention.

At the first visit, we administered a validated questionnaire to evaluate adherence to the MD (scores range from 0 to 18) [14,15]. We developed a personalized, tailored nutrition MD plan agreed upon with the patient to achieve the best compliance. Each woman was provided with a 1-week menu plan and information on the food groups that could be included and those that should be avoided/limited. The energy requirements were calculated for each woman. The diet consisted of 50–55% carbohydrate, 25–30% total fat (≤10% saturated), and 15–20% protein.

Three clinical evaluations were performed during the study: before the start of dietary treatment (T0), three months after the start of the dietary intervention (T1), and after six months from the beginning (T2). At T0, T1 and T2, we evaluated the vitamins’ profile (Folate, B12, B6, E) and zinc, hs-CRP, and Blood redox status (oxidative stress markers such as lipid peroxidation markers, plasma total antioxidant capacity and blood leukocyte subpopulation ROS production).

Oxidative Stress markers were assessed by evaluating ROS (Reactive Species of Oxygen) Assessment in Leukocytes (lymphocytes, monocytes, and granulocytes) by Flow Cytometry Analysis, plasma Lipid Peroxidation Estimation (ALDetect Lipid Peroxidation Assay) (BML-AK170-Enzo Life), Plasma Total Antioxidant Capacity Estimation, ORAC (oxygen radical absorbance capacity) assay, as previously reported [13].

Cardiovascular risk factors and anthropometric parameters were evaluated. Compliance with the MD was evaluated during follow-up visits using a validated questionnaire [15]. Indeed, patients were asked to indicate their pain intensity using the VAS (Visual Analogue Scale). Scores range from 0 to 10. A higher score is more pain (worse).

The investigation was conformed to the principles outlined in the Declaration of Helsinki and approved by the Local Ethics Committee (Azienda Ospedaliero-Universitaria Careggi, Firenze, Italy) (Reference: 21140).

### Statistical Analysis

The sample size was calculated starting from historical data [31] and estimating a reduction in hs-CRP of 0.70 (SD ± 1.4), as previously published [13]. Assuming a power of 0.80 and α setting of 0.05, the number was estimated for 33 women.

The results were expressed as median (range). For categorical variables, chi-square was performed, and the Wilcoxon rank-sum test for continuous variables for paired data. Correlations were assessed using the Spearman method. Differences were considered statistically significant if *p* < 0.05. Statistical analysis was performed using IBM SPSS Statistics 28.0.1.1 for Windows (SPSS, Chicago, IL, USA).

## 5. Conclusions

The results of our study show a clear tendency toward a relationship between pain relief in endometriosis patients and Mediterranean dietary patterns. The tailored Mediterranean dietary intervention appears promising to treat endometriosis-related symptoms. It could be considered a new effective strategy in the long term for chronic pain management in association with other medical interventions.

Adherence to the MD, which includes the consumption each day of vegetables, fruits, whole grains and plant-based fats, a week’s consumption of fish, poultry, legumes, land eggs, moderate portions of dairy products and the limitation of red meat and many foods with added sugar, could help to improve the quality of life and long-term health of young women with endometriosis.

Therefore, the Mediterranean dietary pattern might represent a cornerstone in the gynaecological field.

Further studies are needed to confirm our findings and to deeply explore the role of micronutrients and functional foods, typical of Mediterranean patterns in modulating pathological processes, such as inflammation and oxidative stress, which are responsible for chronic pain in women with endometriosis, also in comparison to other dietary interventions.

## Figures and Tables

**Table 1:** Correlation between Oxidative stress markers and VAS Pain score at T0.

Variables at T0 (n = 35)	VAS Dyspareunia	VAS Non-Menstrual Pelvic Pain	VAS Dyschezia	VAS Dysuria
Lipid peroxidation	-	rho = 0.369 *p* = 0.029	-	rho = 0.416 *p* = 0.013
ORAC	-	rho = −0.394 *p* = 0.019	rho = −0.515 *p* = 0.002	-
M-derived ROS	-	-	-	rho = 0.589 *p* < 0.001
N-derived ROS	-	-	-	rho = 0.607 *p* < 0.001
L-derived ROS	-	-	-	rho = 0.534 *p* = 0.001

N indicates neutrophil; L, lymphocyte; M, monocyte; ROS, reactive oxygen species; ORAC, oxygen radical absorbance capacity.

**Table 2:** Changes in vitamins’ profile from T0 to T2.

Variables	T0	T1	T2	*p* (T0 vs. T1)	*p* (T0 vs. T2)
	(n = 35)	(n = 35)	(n = 26)		
Folate, ng/mL	5.6 (1–14.4)	7.2 (3.9–20)	7.1 (3.7–19.8)	0.01	0.006
Vitamin B12, pg/mL	310 (99–709)	366 (123–760)	336 (199–749)	0.1	0.3
Vitamin E, µg/dL	1285 (720–2612)	1322.5 (889–2828)	1415.5 (952–2615)	0.1	0.1
Zinc, µg/dL	114 (76–193)	125 (89–233)	120 (95–186)	0.1	0.07

Data are reported as median (range).

## Data Availability

Not applicable.
